# Reply to Luppi et al

**DOI:** 10.1093/cid/ciaa324

**Published:** 2020-05-15

**Authors:** John R Wingard, Johan Maertens, Livio Pagano, Sharon C-A Chen, Peter G Pappas, J Peter Donnelly

**Affiliations:** 1 Department of Medicine, University of Florida College of Medicine, Gainesville, Florida, USA; 2 Department of Hematology, University Hospitals Leuven, Leuven, Belgium; 3 Department of Microbiology, Immunology and Transplantation, KU Leuven, Leuven, Belgium; 4 Dipartimento di Diagnostica per Immagini, Radioterapia Oncologica ed Ematologia, Fondazione Policlinico A. Gemelli, IRCCS , Rome, Italy; 5 Sezione di Ematologia, Dipartimento di Scienze Radiologiche ed Ematologiche, Università Cattolica del Sacro Cuore, Rome, Italy; 6 Centre for Infectious Diseases and Microbiology, Laboratory Services, Institute of Clinical Pathology and Medical Research, Westmead Hospital, University of Sydney, Sydney, Australia; 7 Department of Medicine, Division of Infectious Diseases, University of Alabama at Birmingham, Birmingham, Alabama, USA; 8 Department of Hematology, Radboud University Medical Center, Nijmegen, The Netherlands

To the Editor—We thank Dr Luppi and his colleagues for their comments about the mechanisms of ibrutinib contributing to the risk for invasive fungal disease (IFD) [1]. Our manuscript [2] listed the host factors defined as characteristics of individuals clearly predisposed to an IFD. Due to space limitations, we provided no discussion of the mechanism by which such factors contributed to predisposition to IFD.

Our characterization of ibrutinib as a “recognized B-cell immunosuppressant” was not intended to imply that B-cell suppression was the explanation for its association with IFD but rather its primary therapeutic use. The inhibition of Bruton tyrosine kinase (BTK) has become a crucial element in the treatment of indolent B-cell malignancies. Ibrutinib, an irreversible inhibitor of BTK, is now approved for the treatment of chronic lymphocytic leukemia [3], mantle cell lymphoma [4], marginal zone lymphoma [5], small lymphocytic lymphoma [6], and Waldenström macroglobulinemia [7]. BTK has been widely characterized as a critical mediator of B-cell receptor signaling that regulates B-cell survival, activation, differentiation, and interaction with the environment [8]. Hence, BTK is also essential in the development and functioning of adaptive immunity. However, from a host factor’s perspective, the B-cell immunosuppressant activity of ibrutinib is the common denominator for all of these approved therapeutic indications. BTK also plays a major role in innate immunity (1) in Toll-like receptor–mediated recognition of infectious agents; (2) in maturation, recruitment, and function of innate immune cells, including neutrophils, monocytes, macrophages, and T cells; and (3) in regulating NLRP_3_ inflammasome activation [9].

We agree that ibrutinib has multiple effects on immunity other than B-cell activity, including activity against T cells, macrophages, and multiple inflammatory pathways, which makes it an effective second therapy for graft-vs-host disease after failure of one or more lines of systemic therapy [10].

## Notes

***Potential conflicts of interest.*** The authors: No reported conflicts of interest. All authors have submitted the ICMJE Form for Disclosure of Potential Conflicts of Interest.
